# Metal-organic framework-based nanomaterials for CO_2_ storage: A review

**DOI:** 10.3762/bjnano.14.79

**Published:** 2023-09-20

**Authors:** Ha Huu Do, Iqra Rabani, Hai Bang Truong

**Affiliations:** 1 VKTech Research Center, NTT Hi-Tech Institute, Nguyen Tat Thanh University, Ho Chi Minh City 700000, Vietnam; 2 Department of Nanotechnology and Advanced Materials Engineering, Sejong University, Seoul 05006, Republic of Korea; 3 Optical Materials Research Group, Science and Technology Advanced Institute, Van Lang University, Ho Chi Minh City, Vietnam; 4 Faculty of Applied Technology, School of Technology, Van Lang University, Ho Chi Minh City, Vietnam

**Keywords:** CO_2_ storage, metal-organic frameworks, nanomaterials, open metal sites, pore size

## Abstract

The increasing recognition of the impact of CO_2_ emissions as a global concern, directly linked to the rise in global temperature, has raised significant attention. Carbon capture and storage, particularly in association with adsorbents, has occurred as a pivotal approach to address this pressing issue. Large surface area, high porosity, and abundant adsorption sites make metal-organic frameworks (MOFs) promising contenders for CO_2_ uptake. This review commences by discussing recent advancements in MOFs with diverse adsorption sites, encompassing open metal sites and Lewis basic centers. Next, diverse strategies aimed at enhancing CO_2_ adsorption capabilities are presented, including pore size manipulation, post-synthetic modifications, and composite formation. Finally, the extant challenges and anticipated prospects pertaining to the development of MOF-based nanomaterials for CO_2_ storage are described.

## Introduction

One of the major issues associated with CO_2_ emissions is the heightened risk of climate change faced by our planet. Furthermore, there is an alarming issue of elevated levels of air pollution affecting human population [1]. One potential solution to address this problem involves increased government funding for the maintenance of existing renewable energy sources and the development of new green energies [2–3]. However, the widespread implementation of these solutions is hindered by the challenges posed by nascent technology [4–5]. Another feasible approach to tackle this issue is the advancement of carbon capture and storage (CCS) methods, particularly those involving highly efficient adsorbents [6–7]. The CCS process has the capability to effectively treat substantial volumes of CO_2_ emissions originating from conventional fossil fuel sources [8–10]. Therefore, identification and development of durable and efficient adsorbents are critical to the successful implementation of CCS. Until now, various classes of materials have been investigated for CO_2_ adsorption, such as covalent organic frameworks, molecular sieves, activated carbon, and metal-organic frameworks (MOFs) [11–13]. Notably, MOFs constructed from metal ions and organic linkers are expected to be alternative materials to the organic alcohol amines in CCS [14]. These nanosized materials posess unique properties such as ultrahigh surface area, tunable pore size, open metal sites (OMSs), and facile post-synthetic modifications, which allow for diverse strategies towards efficient adsorption and separation of gas molecules [15]. Among the nanosized MOFs, MOF-210 has demonstrated a remarkable ability to adsorb CO_2_ (54.5 mmol·g^−1^ at 50 bar, 298 K) owing to its large surface area of 6240 m^2^·g^−1^ [16–19]. However, several studies have indicated that the surface area is not the sole determining factor in CO_2_ storage at low pressure. For instance, despite having a lower surface area than MOF-177, HKUST-1 exhibited a greater CO_2_ adsorption capacity of 4.16 mmol·g^−1^ at 298 K and 1 bar [20]. This discrepancy can be attributed to the presence of unsaturated Cu metal centers within the MOF structure, which facilitate interactions with CO_2_ molecules. Additionally, the MOF-74 family, built from metal(II) oxide chains linked by 2,5-dioxido-1,4-benzenedicarboxylate, exhibited a high CO_2_ adsorption ability with OMSs ranging from 5.5 to 8.0 mmol·g^−1^ at 296 K and 1 bar [21]. Moreover, acid Lewis sites within these MOFs effectively interact with CO_2_, leading to increased adsorption. The pore size plays a vital role in the adsorption and separation of CO_2_ from gas mixtures because of the different kinetic diameters of gas molecules.

Herein, we present a comprehensive examination of the current scientific literature pertaining to the utilization of metal-organic framework (MOF)-based nanomaterials in the context of CO_2_ storage and conversion. This account focuses on the introduction of MOFs featuring chemical sites, such as open metal sites (OMSs) and Lewis acid sites, for CO_2_ adsorption applications. Furthermore, we explore several approaches that have been employed to enhance CO_2_ storage capabilities, including pore size control, post-synthetic modification, and the development of composites. Finally, the expected direction and existing challenges in progressing MOF-based nanomaterials for CO_2_ storage are discussed.

## Review

### Nanosized MOFs with adsorption centers for CO_2_ capture

#### MOFs with open metal sites

OMSs are a fundamental characteristic of MOF-based nanomaterials, generating strong interactions with other chemical species. Extensive literature has been devoted to the investigation of MOFs with OMSs. Notably, HKUST-1, featuring copper OMSs, displayed a CO_2_ uptake of 4.1 mmol·g^−1^ at 298 K and 1 bar [22]. The effectiveness of OMSs has been exemplified in a study conducted by Caskey and coworkers [21]. Under conditions of 296 K and 1 bar, the authors observed that Mg-MOF-74 exhibited the highest CO_2_ absorption capacity, reaching 8.0 mmol·g^−1^, followed by Co-MOF-74 with 7.0 mmol·g^−1^. In contrast, Zn-MOF-74 demonstrated the lowest performance, displaying an adsorption capacity of 5.5 mmol·g^−1^. This discrepancy was attributed to the shorter length of the Mg–O bonds, which facilitated enhanced electrostatic interactions between the Mg sites and CO_2_ molecules. Moreover, Mg-MOF-74 exhibited a higher heat of adsorption than other variants. Kim et al. synthesized bimetallic MOFs, specifically Mg/Zn-MOF-74 and Ni/Zn-MOF-74, for CO_2_ storage [23]. The presence of different metal ions in bimetallic MOFs generated a synergistic effect, leading to a higher CO_2_ adsorption capacity compared to Zn-MOF-74.

**Figure 1:**
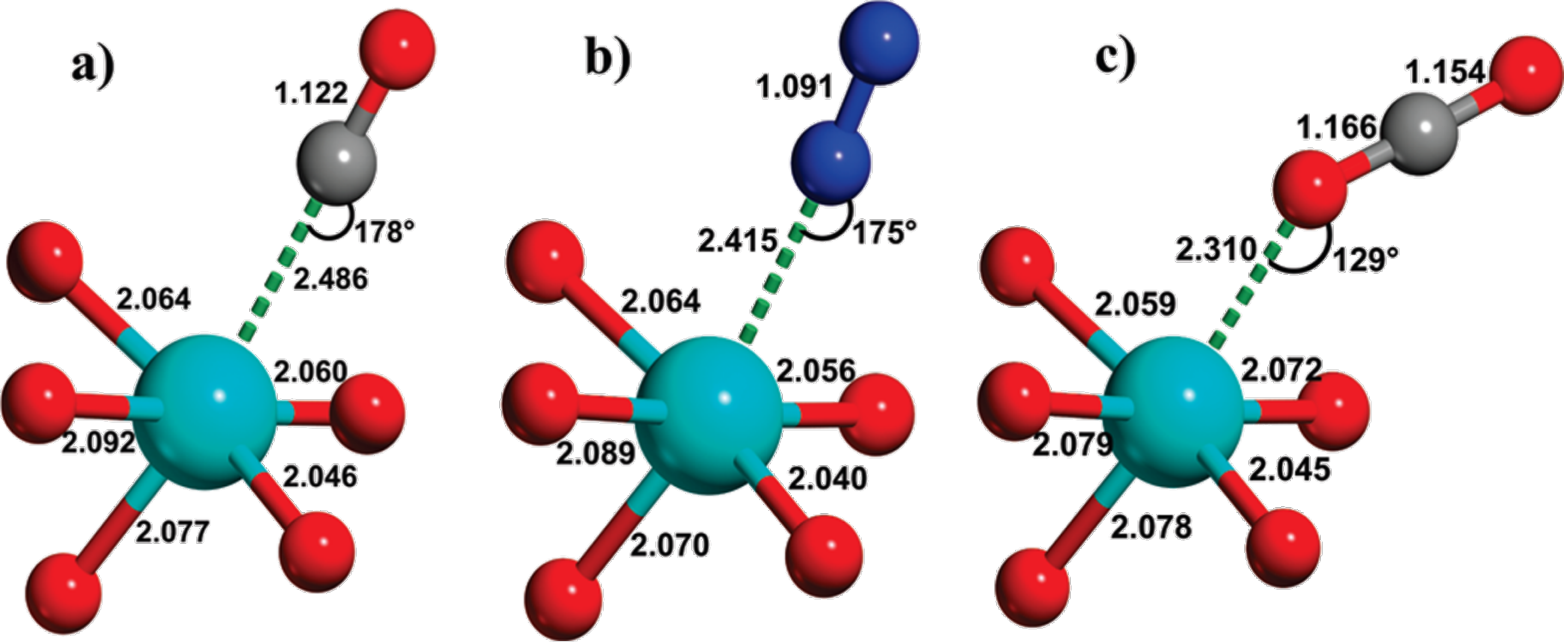
Distances (Å) and angles of interaction between Mg-MOF-74, [Mg/DOBCD] (DOBCD: 2,5-dioxido-1,4-benzenedicarboxylate), with (a) CO, (b) N_2_, and (c) CO_2_. Figure 1 was reprinted with permission from [25]. Copyright 2010 American Chemical Society. This content is not subject to CC BY 4.0.

Also, several theoretical computations have been implemented to elucidate the adsorption mechanism of CO_2_ on MOFs featuring OMSs. Wu et al. revealed that the interactions between the OMSs of Mg-MOF-74 and HKUST-1 and CO_2_ molecules are primarily of physical nature [24]. This type of adsorption mechanism offers the advantage of low energy requirements in material regeneration. Another significant contribution in the field of mechanism studies was made by Valenzano and coworkers [25]. The recorded an adsorption angle of 129° for CO_2_ adsorption on Mg-MOF-74, which is smaller than the corresponding angles observed for N_2_ and CO, implying a stronger interaction between Mg-MOF-74 and CO_2_ (Figure 1).

#### MOFs with Lewis basic centers

**Figure 2:**
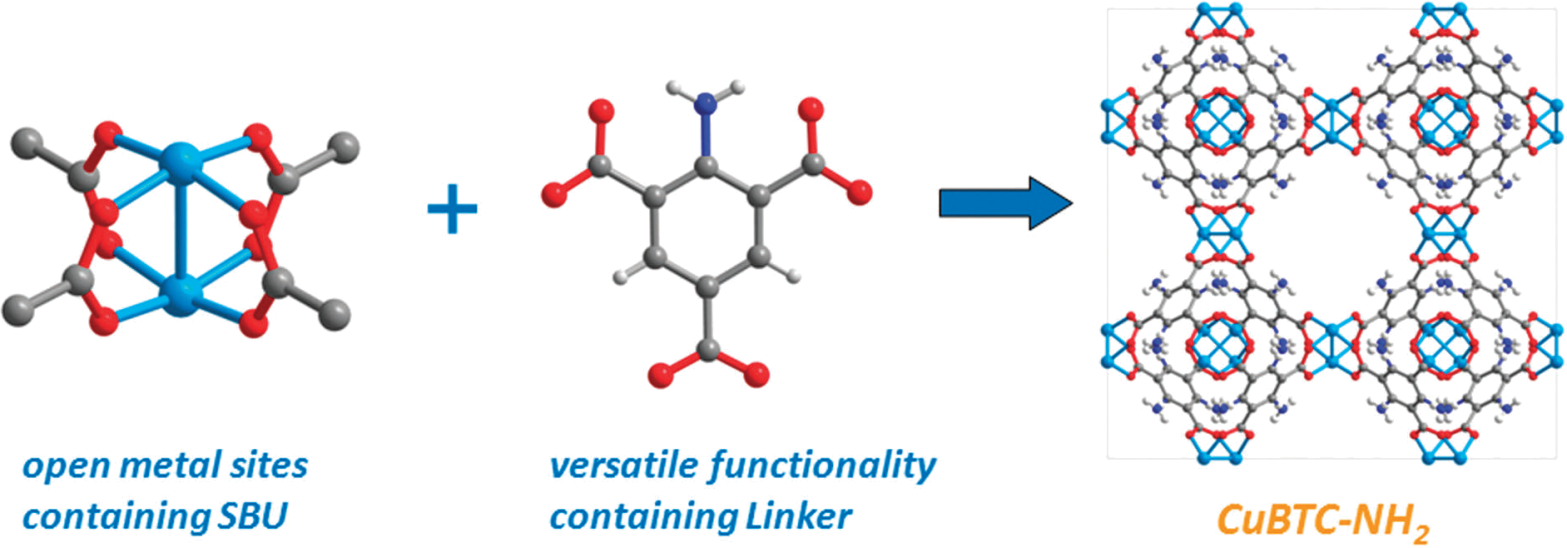
Schematic illustration for the preparation of a CuBTC-NH_2_ nanomaterial. Figure 2 was republished with permission of The Royal Society of Chemistry, from [27] (“Amino substituted Cu_3_(btc)_2_: a new metal–organic framework with a versatile functionality” by K. Peikert et al., Chem. Commun., vol. 48, © 2012); permission conveyed through Copyright Clearance Center, Inc. This content is not subject to CC BY 4.0.

N-containing linkers serve as secondary units in the construction of MOFs with Lewis basic centers (LBCs), producing strong interactions with CO_2_ gas [26]. An illustrative example of this is demonstrated by Peikert et al., who employed 1,3,5-benzenetricarboxylic acid (BTC) to prepare Cu-based MOF nanoparticles (UHM-30) for gas storage, as depicted in Figure 2 [27]. The benefit of this strategy is the generation of both OMSs and LBCs, resulting in an enhanced CO_2_ adsorption capacity for UHM-30 (5.26 mmol·g^−1^) compared to HKUST-1 (4.69 mmol·g^−1^). The effectiveness of amino groups in facilitating CO_2_ adsorption has been further demonstrated by Si and coworkers [28]. The authors used 2-amino-1,4-benzenedicarboxylate to fabricate an amine-containing MOF, which exhibited a high CO_2_ adsorption capacity of 7.2 mmol·g^−1^. Moreover, MOFs constructed by N-containing aromatic ring linkers have been examined for CO_2_ capture. For instance, Shimizu et al. used 3-amino-1,2,4-triazolate as a linker to create Zn-based MOF nanomaterials, yielding a high efficiency in CO_2_ storage [29]. The result was attributed to the favorable interaction between CO_2_ and NH_2_ groups. Likewise, Panda utilized 5-aminotetrazole to synthesize ZTF-1, a MOF nanomaterial featuring LBCs [30]. This approach offered the advantage of creating a synergistic effect, leading to a noteworthy CO_2_ adsorption capacity of 5.6 mmol·g^−1^.

### Strategies for enhanced CO_2_ storage in MOF-based nanomaterials

#### Pore size control

Individual MOFs displayed promising capacities for CO_2_ adsorption. Nevertheless, to meet industrial criteria, it is necessary to enhance the CO_2_ capture performance of MOFs. Therefore, various methods were applied to modify the physical and chemical properties of MOFs, leading to improved performance. The manipulation of pore size by modifying the size of the ligands is an effective approach for accelerating CO_2_ adsorption. For instance, Yao et al. used different ligands to fabricate various Zn-based MOF nanomaterials with distinct pore volumes for CO_2_ adsorption applications [31]. Notably, SUMOF-2, characterized by the smallest pore diameter (5.9 Å), displayed the highest CO_2_ adsorption capacity at 273 K. The interpenetrated linkers contribute to the expansion of the cavity and the creation of an electric field gradient, enhancing the affinity for CO_2_. Prasad et al. employed 4-(2-carboxyvinyl)benzoic acid to create nanosized SNU-70, which possesses a pore size of 9 Å, whereas SNU-71, with a 4-(2-carboxyethyl)benzoic acid linker has a pore size of 2.5 Å [32]. The outcomes revealed that SNU-71 displayed a greater CO_2_ uptake than SNU-70, attributing to its smaller pore size.

**Figure 3:**
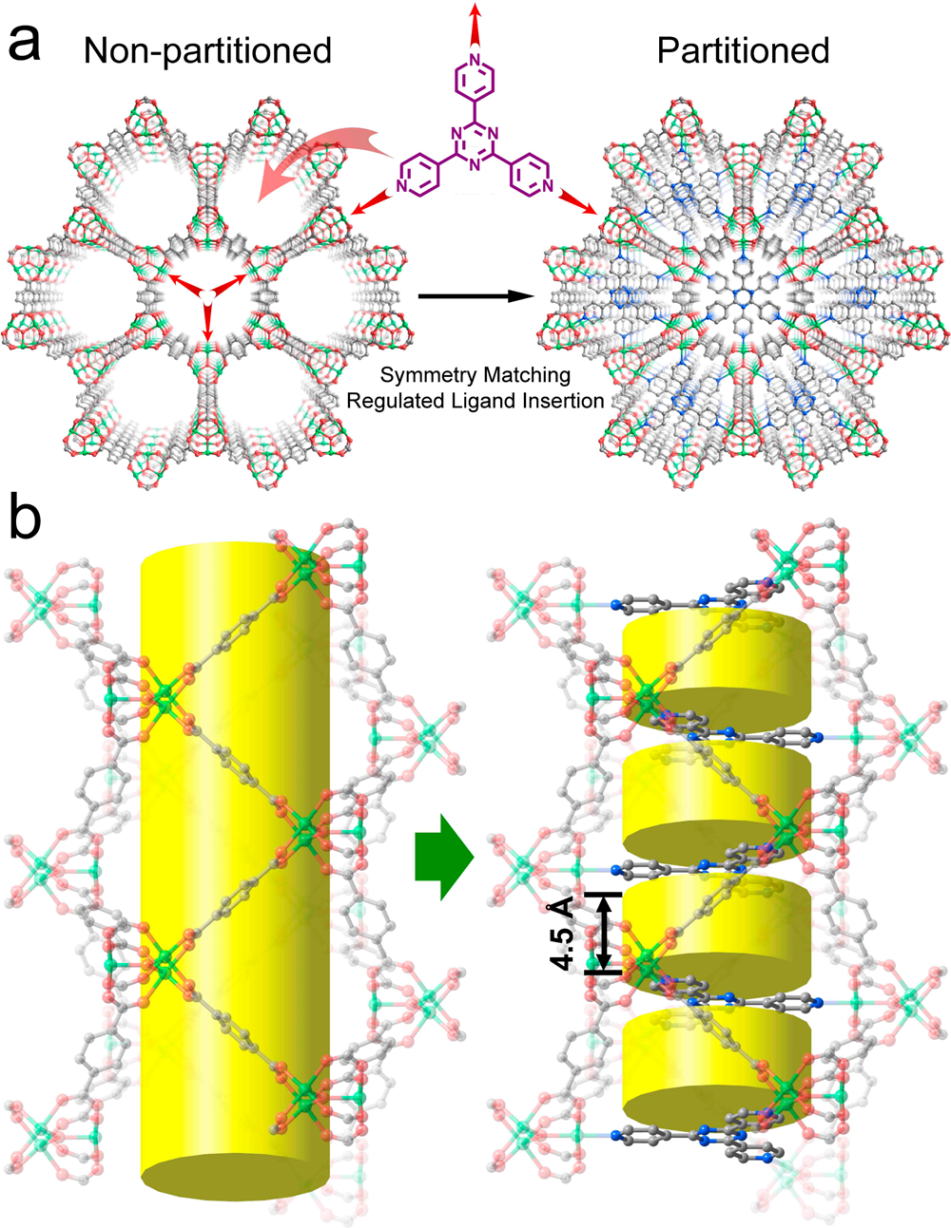
Graphic illustration of pore space partition by inserting TPT in MIL-88 structure. (a) View along the *c* axis and (b) side view of the channels displaying the cylindrical pores. Figure 3 was reprinted with permission from [34]. Copyright 2015 American Chemical Society. This content is not subject to CC BY 4.0.

Pore space partition (PSP) is an intriguing strategy for enhancing gas storage and gas separation, encompassing species such as CH_4_, CO_2_, and N_2_. This approach involves the introduction of additional linkers to divide large pores into smaller compartments [33]. PSP not only offers an increased number of active sites but also enhances the efficiency of the cavity space. To exemplify this, Zhao et al. used a 2,4,6-tri(4-pyridyl)-1,3,5-triazine linker to split the pores of MIL-88, as shown in Figure 3 [34]. Consequently, a substantial CO_2_ adsorption capacity of 5.6 mmol·g^−1^ was recorded at 273 K and 1 bar, comparable to that of MOF-74 under the same conditions. The efficacy of the pore space partition strategy has also been indicated in a report of Zheng and coworkers [35]. By combining building units of [In(CO_2_)_4_]^−^ and [In_3_O]^+^, a core–shell kind of In-based MOF was created, exhibiting a remarkable CO_2_ adsorption capacity of approximately 3.6 mmol·g^−1^ at 273 K and 1 bar.

#### Post-synthetic functionalization

**Figure 4:**
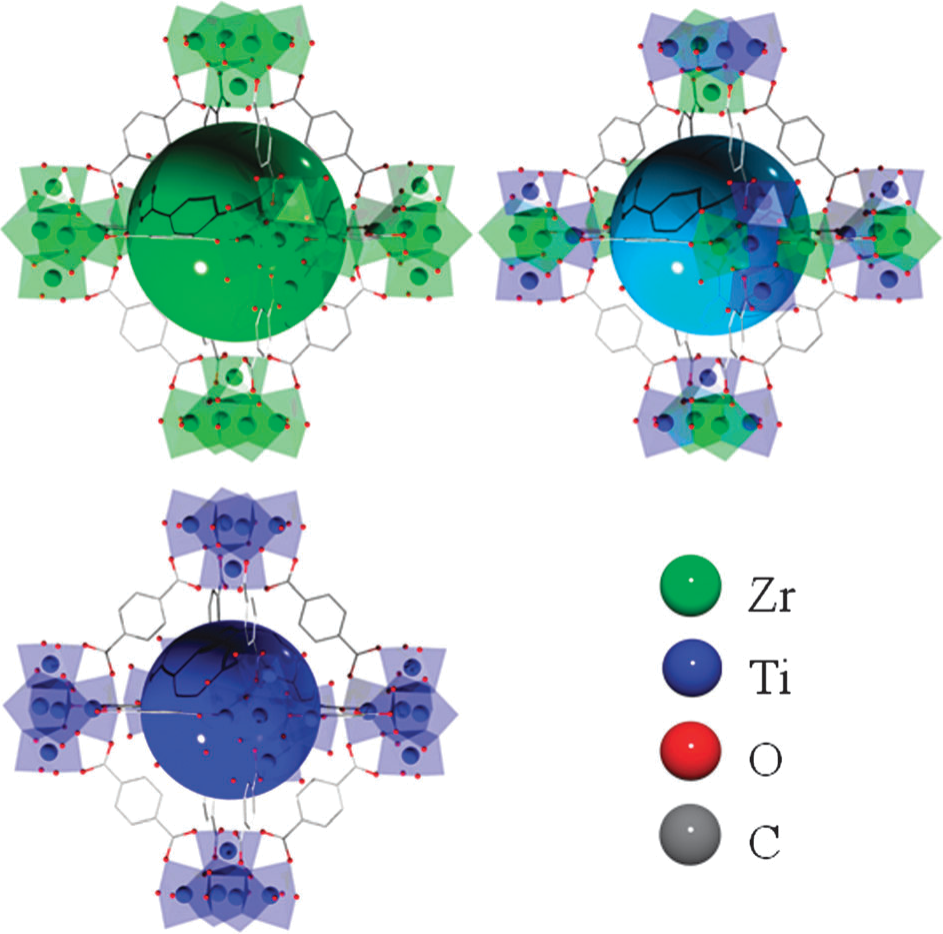
Graphic illustration of the ion exchange by Ti(IV) in UiO-66. Figure 4 was republished with permission of The Royal Society of Chemistry, from [38] (“A route to drastic increase of CO_2_ uptake in Zr metal organic framework UiO-66” by C. H. Lau et al., Chem. Commun., vol. 49, © 2013); permission conveyed through Copyright Clearance Center, Inc. This content is not subject to CC BY 4.0.

Amino groups, which have been widely recognized as LBCs in MOFs for the purpose of capturing CO_2_, can be grafted onto MOFs through a post-synthetic process. Several studies adopted this strategy to modify the properties of MOFs for gas storage. For instance, Zhang et al. used ethylenediamine to functionalize ZIF-8, resulting in enhanced CO_2_ adsorption and selectivity [36]. A benefit of this approach is that the surface area was improved (nearly 40%), while ED-ZIF-8 yielded a two-fold higher amount of adsorbed CO_2_ than pure ZIF-8. In a recent study, Gaikwad et al. modified MOF-177 nanoparticles using three different amines to enhance the amount of absorbed CO_2_ [37]. The optimally functionalized MOF-177, employing tetraethylenepentamine (TEPA), exhibited a remarkably higher amount of absorbed CO_2_ than bare MOF-177 under the same conditions. This enhancement was attributed to the favorable distribution of the relatively small-sized TEPA within the pore space of MOF-177, allowing for more favorable interactions between CO_2_ molecules and the amine centers. It is noteworthy that modifications to MOFs have not been limited to the linker molecules but have also involved the metal ions. For example, Lau et al. used an ion exchange strategy to boost the amount of adsorbed CO_2_ in UiO-66 (Figure 4) [38]. After replacing approximately 50% of the Zr^4+^ ions in UiO-66 with Ti^4+^, a significant increase in CO_2_ adsorption capacity (81%) was achieved. Several explanations can be considered for this observation. First, the substitution of heavy metal ions (Zr^4+^) with lighter ones (Ti^4+^) could result in an increased specific surface area of UiO-66 (BET surface area: 1844 m^2^·g^−1^). Second, the shorter Ti–O bond lengths compared to Zr–O bonds lead to a reduced pore size in UiO-66(Zr/Ti), thereby facilitating improved interactions between the MOF and CO_2_ molecules.

#### MOF composites

**Figure 5:**
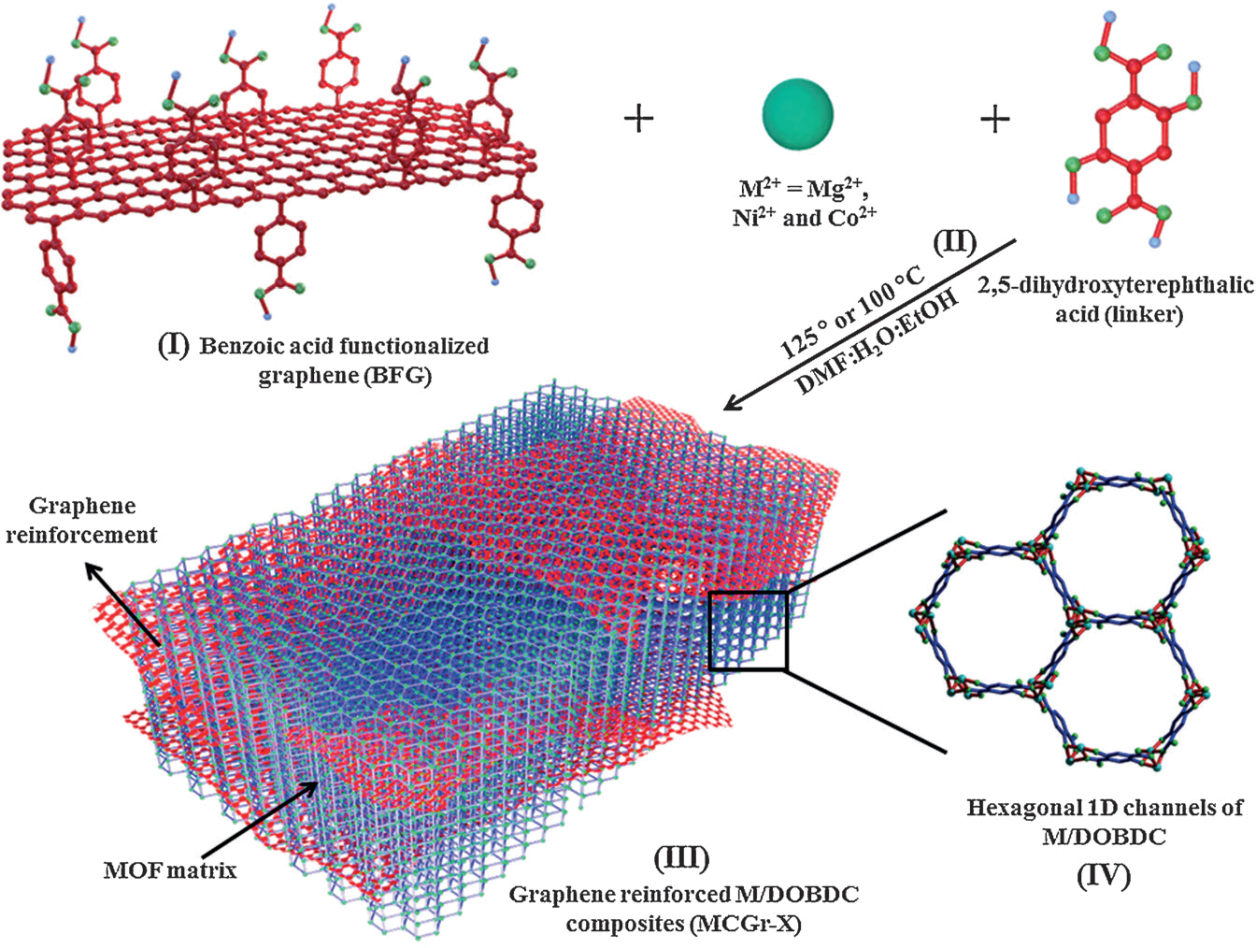
Graphic illustration of the fabrication of MOF/graphene-based composites. Figure 5 was reproduced from [41], R. Kumar et al., “Remarkable Improvement in the Mechanical Properties and CO_2_ Uptake of MOFs Brought About by Covalent Linking to Graphene”, Angew. Chem., Int. Ed., with permission from John Wiley and Sons. Copyright © 2016 WILEY-VCH Verlag GmbH & Co. KGaA, Weinheim. This content is not subject to CC BY 4.0.

Post-synthetic functionalization was recognized as an effective strategy for enhancing the adsorption ability of nanosized MOF structures. However, it demands chemical sites or an appropriate pore size for agent insertion. Many MOFs lack these properties, rendering them unsuitable for post-synthetic strategies. Fortunately, the development of MOF composites is a promising solution to augment the quantity of CO_2_ that can be absorbed. As a case in point, Eshraghi et al. fabricated various composites of Cr- and Cu-based nanosized MOFs and multiwalled carbon nanotubes (MWCNTs) [39]. The authors found that the amount of absorbed CO_2_ increased significantly by 64% (at 298 K and 18 bar) for MIL-100(Cr) following modification. Similarly, for Cu_3_(BTC)_2_, there was a dramatic growth of 3.46 mmol·g^−1^ in the amount of absorbed CO_2_ after the modification, ascribed to the improved pore space by MWCNTs. In another work, MOF/carbon-based composites were reported by Liu and coworkers [40]. The authors used graphene oxides as templates for growing Cu-MOF nanograins for gas storage. A benefit of this strategy was that surface area was enhanced, resulting in a significant 30% increase in the amount of CO_2_ adsorbed for the optimal Cu-MOFs/GO composites. Likewise, Kumar et al. employed graphene-based materials to reinforce MOF structures for the improvement of CO_2_ uptake [41]. The authors used carboxylic acids to modify graphene nanolayers, and then performed in situ synthesis of different MOF-74 materials on the graphene matrix via a solvothermal method (Figure 5). Although the surface area of composites was only slightly increased compared to the initial materials, the amount of absorbed CO_2_ was significantly improved. Notably, the optimal Mg-MOF-74/graphene-based composite yielded a high CO_2_ adsorption capacity of 8.4 mmol·g^−1^ at 298 K and 1 bar.

### Grand canonical Monte Carlo simulation for CO_2_ storage prediction

Grand canonical Monte Carlo (GCMC) simulation is an effective method to predict the gas adsorption ability of porous materials. For instance, Tao et al. used the GCMC method to evaluate CO_2_ adsorption on various materials, including MOF-5, ZIF-8, and Mg-MOF-74 [42]. The authors found that the computational results were in agreement with experimental investigations under 10 bar and 298 K. At pressures higher than 10 bar, computational and experimental results were significantly different because of the unsuitable force field under high pressure conditions. Recently, Stanton et al. also used the GCMC technique to predict the CO_2_ adsorption of various MOF structures that were modified with amino groups [43]. This study could provide adequate information for further experiments in CO_2_ storage areas.

## Conclusion

Undoubtedly, MOFs have been widely recognized as promising nanomaterials for CO_2_ adsorption, offering potential mitigiation of the impact of CO_2_ gas on global warming. However, despite the considerable research efforts dedicated to MOFs in the context of CO_2_ adsorption, certain drawbacks still need to be addressed. Primarily, a large number of MOFs have poor CO_2_ selectivity in the presence of water and acid gases. These species have higher polarities, which promotes their interaction with OMSs within the MOF structures. In this regard, MOFs tend to exhibit instability under such conditions. Therefore, design and selection of MOFs are imperative for effective CO_2_ adsorption, such as in the case of MOF-808. Furthermore, CO_2_ storage at low pressure is still limited due to poor CO_2_/MOF interaction. Although this challenge can be addressed by modifying the ligand components of MOFs with hydrophilic groups, such modifications result in a substantial increase in the cost of adsorbent production. Finally, it is crucial to explore low-cost and environmentally friendly methods for the synthesis of nanosized MOFs to cater to specific applications.
